# Quantitative gait assessment in children with 16p11.2 syndrome

**DOI:** 10.1186/s11689-019-9286-9

**Published:** 2019-10-27

**Authors:** Sylvie Goldman, Aston K. McCullough, Sally Dunaway Young, Carly Mueller, Adrianna Stahl, Audrey Zoeller, Laurel Daniels Abbruzzese, Ashwini K. Rao, Jacqueline Montes

**Affiliations:** 1 Department of Neurology, Division of Child Neurology, Columbia University Irving Medical Center, Presbyterian Hospital, 622 W 168th Street, PH18-331, New York, NY 10032 USA; 2 G.H. Sergievsky Center, Columbia University Irving Medical Center, Presbyterian Hospital, 622 W 168th Street, New York, NY 10032 USA; 3 Department ot Biobehaviroal Science, Columbia University, Teacher College, 525 West 120th Street, New York, NY 10027 USA; 4 Department of Neurology, Division of Neuromuscular Medicine, Stanford University, 2652 East Bayshore Road, Palo Alto, CA 94303 USA; 5 Department of Rehabilitation and Regenerative Medicine, Programs in Physical Therapy, Columbia University Irving Medical Center, Presbyterian Hospital, 622 W 168th Street, New York, NY 10032 USA

**Keywords:** Gait, Motor function, 16p11.2, Children, Neurodevelopment disorder, Autism spectrum disorder

## Abstract

**Background:**

Neurodevelopmental disorders such as 16p11.2 syndrome are frequently associated with motor impairments including locomotion. The lack of precise measures of gait, combined with the challenges inherent in studying children with neurodevelopmental disorders, hinders quantitative motor assessments. Gait and balance are quantifiable measures that may help to refine the motor phenotype in 16p11.2. The characterization of motor profile is useful to study the trajectories of locomotion performance of children with genetic variants and may provide insights into neural pathway dysfunction based on genotype/phenotype model.

**Methods:**

Thirty-six children (21 probands with 16p11.2 deletion and duplication mutation and 15 unaffected siblings), with a mean age of 8.5 years (range 3.2–15.4) and 55% male, were enrolled. Of the probands, 23% (*n* = 6) had a confirmed diagnosis of autism spectrum disorder (ASD) and were all male. Gait assessments included 6-min walk test (6MWT), 10-m walk/run test (10MWR), timed-up-and-go test (TUG), and spatio-temporal measurements of preferred- and fast-paced walking. The Pediatric Evaluation of Disability Inventory-Computer Adaptive Tests (PEDI-CAT), a caregiver-reported functional assessment, was administered. Measures of balance were calculated using *percent time in double support* and *base of support*. Analyses of the six children with ASD were described separately.

**Results:**

Thirty-six participants completed the protocol. Compared with sibling controls, probands had significantly lower scores on the 6MWT (*p* = 0.04), 10MWR (*p* = 0.01), and TUG (*p* = 0.005). Group differences were also identified in *base of support* (*p* = 0.003). Probands had significantly lower PEDI-CAT scores in all domains including the mobility scale (*p* < 0.001). Using age-matched subsamples, the ASD and non-ASD genetic variant groups had larger *base of support* compared to the controls. In the fast-paced condition, all participants increased their velocity, and there was a corresponding decrease in *percent time in double support* compared to the preferred-pace condition in all participants. Only the ASD group presented with upper limb arm/hand stereotypies.

**Conclusions:**

Children with 16p11.2, with and without ASD, present with balance impairment during locomotion activities. Probands performed worse on functional assessments, and quantitative measures revealed differences in *base of support.* These results highlight the importance of using precise measures to differentiate motor dysfunction in children with neurodevelopmental disorders.

## Background

Neurodevelopmental disorders are characterized by a wide range of impairments with broad severity level. Among the affected developmental functions, motor impairments represent the earlier and most visible signs [1, 2]. Yet, the motor phenotypes are often difficult to quantify precisely in children with neurodevelopmental disorders especially when accompanied by high behavioral comorbidities such as those with autism spectrum disorder (ASD) [3] who may display low compliance, distractibility, and poor imitation skills. These behavioral challenges often confound traditional command-based motor assessments. With recent technological advances, many gene-based syndromes have been identified that provide ways to reduce phenotypic heterogeneity [4, 5]. Focusing on a homogenous cohort of children with selected genetic conditions such as 16p11.2, the most frequent etiologies for ASD [2, 6–8] have led to fruitful genotype/phenotype findings [9–12]. Children with 16p11.2 mutation (deletion or duplication) present with a range of neurodevelopmental impairments affecting mostly cognitive (i.e., language) and behavioral (attention deficit, autism spectrum disorder) and motor (i.e., coordination) functions [13–15]. The prevalence of comorbid diagnosis of autism spectrum disorder in individuals with 16p11.2 deletion has been estimated between 20 and 33% [14, 16]. Speech articulation, limb and trunk hypotonia, abnormalities of agility, and seizures have been reported in a large cohort of carriers with both 16p11.2 deletion and duplication [17]. Interestingly, individuals with 16p11.2 deletion present more commonly with macrocephaly, whereas individuals with 16p11.2 duplication present with microcephaly. Overall, studies suggest children with duplications present with milder impairments [18]. In addition to the neurological phenotypes, studies have reported an unexplained high prevalence of obesity [19], which may be related to level of physical activity. Despite the high prevalence of motor impairment in children with 16p11.2 with and without ASD, the majority of studies so far have relied on parent questionnaires to study the motor profile [17, 20]. The lack of quantitative performance measures, combined with the inherent challenges in studying children with neurodevelopmental disorders, hinders objective motor assessments. Yet, children with genetic variants such as 16p11.2 constitute cohorts with homogenous etiologies that allow for focused motor assessment to offer insights into neural pathway dysfunction such as the cerebellum or the basal ganglia, responsible for motor abnormalities, and that have been identified in other neurodevelopmental disorders such as ASD [21, 22].

In this descriptive study, we used an instrumented walkway and a battery of standardized quantitative measures of locomotion (gait) and mobility (functionality) to compare 16p11.2 probands with and without a diagnosis of ASD to a group of non-affected siblings.

## Methods

Children included in this study were recruited as part of a large research family meeting that provided the genetic (16p11.2 deletion/duplication) and behavioral diagnosis (ASD) based on prior evaluations. Informed consent was obtained from all individual participants’ caregivers included in the study. The experimental protocol conformed to the Institutional Review Board of Columbia University Irving Medical Center and followed their ethical guidelines. Thirty-six children (21 probands with 16q11.2 mutation, deletion (*n* = 18), or duplication (*n* = 3) and 15 unaffected siblings), with a mean age of 8.5 years (range 3.2–15.4), 55% male, and 23% incidence of ASD, completed the study. Measures previously used in other pediatric neurodevelopmental and neuromuscular disorders were used to characterize motor function [23–26]. Gait assessments included 6-min walk test (6MWT), 10-m walk/run test (10MWR), timed-up-and-go test (TUG), and spatio-temporal measurements of preferred- and fast-paced gait. Children were required to walk in 10-m bouts across the GAITRite™ instrumented walkway to calculate temporal and spatial gait parameters (velocity and stride length). Measures of balance using *percent time in double support* (%) and *base of support* (cm) from the walkway were collected during these assessments. Subsequently, gait data from the six children diagnosed with ASD were compared with six age-matched non-ASD genetic variant and six non-ASD sibling controls. The Pediatric Evaluation of Disability Inventory-Computer Adaptive Test (PEDI-CAT) [27], a caregiver-questionnaire with a long history of application in developmental medicine, was administered as a measure of daily living motor functionality. The PEDI-CAT is comprised of three functional domains: Daily Activities, Mobility, and Social/Cognitive, and an additional Responsibility domain related to the extent to which the caregiver or child takes responsibility for managing complex, multi-step life tasks. Questions in the (1) Daily Activities domain relate to household maintenance (e.g., eating, dressing); (2) the Mobility domain evaluates the child ability to move in different environments such as in home and at school (e.g., standing, running); (3) the Social/Cognitive domain includes an assessment of communication, interaction, safety behavior, play, attention, and problem solving; and (4) the Responsibility domain considers the extent to which the child is able to seek assistance as needed and direct others in order to accomplish tasks that enable independent living.

### Statistical analyses

Data were analyzed in MATLAB R2017a [28]. To account for age, gender, and height differences, distance walked in meters on the 6MWT was expressed as a percent of predicted based on published norms [29]. Descriptive statistics are shown as median (interquartile range) and frequencies [% (*n*)]. Given the sibling-matched control design used in this study, the assumption of independence was expressly assessed for all outcome measures of interest using Spearman’s correlations within clusters of sibling pairs (*n* = 10). All measures included in the following analyses met the requisite assumption of independence (*p* > 0.05). Associations between PEDI-CAT subdomain scores and all gait and functional assessment scores were also separately evaluated within each group (i.e., children with and without the genetic variant) using Spearman correlation coefficients. Preliminary analyses revealed that gait parameters tended to be non-normally distributed in this sample. Resultantly, differences in gait parameters between children with genetic variant 16p11.2 and their siblings were tested using a Wilcoxon rank sum test, and the rank sum test statistic (*W*) was reported. The Wilcoxon rank sum test is appropriate for detecting differences in medians between two independent samples, especially when data do not follow a normal distribution [30]. All models met the assumptions of the tests, and the significance level was set at *α* = 0.05.

## Results

Clinical characteristics of children with and without genetic variant 16p11.2 are shown in Table 1. Proband children had longer performance times on the TUG (*W* = 408; *p* = 0.009) and 10MWR (*W* = 460; *p* = 0.02) and had lower percent of predicted distances (i.e., walked shorter distance) on the 6MWT (*W* = 230; *p* < 0.001) than children without the genetic variant. As shown in Table 2, probands had a wider *base of support* in both walking conditions (*W* = 478; *p* = 0.004). Probands had significantly lower scores in all domains of the PEDI-CAT, including the Mobility subscale compared to siblings (*W* = 287; *p* = 0.001). In proband children, caregiver-reported scores for Daily Activity subscale were positively associated with the 6MWT (*ρ* = 0.49, *p* < 0.05). There was no association in proband children and siblings between caregiver reported scores on PEDICAT subscales (Daily Activities, Mobility, Social/Cognitive, and Responsibility) and other quantitative measures of gait (6MWT percent of predicted distance, 10MWR, and *base of support*) and function (TUG) (*p* > 0.05).

**Table 1:** Characteristics of proband children with 16p11.2 and their unaffected siblings

Variable	Genetic variant (*n* = 21)	Unaffected siblings (*n* = 15)
Sex (*n*)		
Female	11	6
Age	9.3 (5.4)	7.9 (7.8)
BMI percentiles^a^, % (*n*)		
Underweight	10% (2)	7% (1)
Normal weight	33% (7)	73% (11)
Risk for overweight	24% (5)	13% (2)
Overweight	33% (7)	7% (1)
PEDI-CAT		
Daily Activities	34 (20.5)	47 (9.3)
Mobility	34 (26.8)	49 (11.3)
Social/Cognitive	34 (13.8)	46 (6.8)
Responsibility	39 (13.0)	50 (14.3)

Table values are medians (interquartile range) and frequencies % (*n*)

*Abbreviations*: *BMI* body mass index, *PEDI-CAT* Pediatric Evaluation of Disability Inventory-Computer Adaptive Test normative standard scores

^a^Centers for Disease Control BMI percentiles (Ogden 2002 [31]).

**Table 2:** Differences in gait parameters between proband children with 16p11.2 and their unaffected siblings

Variable	Genetic variant	Unaffected siblings
Timed-up-and-go test (sec)	4.81 (1.4)	4.19 (1.3)*
10-m walk/run test (sec)	3.52 (1.0)	2.91 (0.7)*
6-min walk test (meters)	429 (72.5)	496 (201.3)
6-min walk test (% predicted)	71.6 (11.6)	88 (11.1)***
Preferred-pace walking		
Velocity (cm/sec)	114.5 (18.4)	130.4 (31.6)
Stride length (cm)	112.5 (24.0)	110.6 (49.2)
Base of support (cm)	10.3 (4.7)	6.9 (2.4)**
Double support time (%)	22.9 (6.1)	20.0 (4.7)
Fast-pace walking		
Velocity (cm/sec)	198.8 (50.1)	192.7 (55.0)
Stride length (cm)	133.8 (36.0)	127.7 (65.6)
Base of support (cm)	10.0 (5.1)	6.7 (2.4)*
Double support time (%)	14.9 (5.6)	11.9 (7.2)

Significant differences in gait parameters between children with and without genetic variant 16p.11.2. Descriptive values are *medians* (interquartile range)

*Abbreviations*: *m* meter, *min* minute, *cm* centimeters, *sec* seconds

*p* < 0.05 (*), *p* < 0.01 (**), *p* < 0.001(***)

## Discussion

For the first time, this study reports the results of a detailed battery of gait assessments to characterize motor performance in children with 16p11.2 deletion or duplication. We found that probands have reduced performance on functional motor tasks and lower endurance compared to their non-affected siblings. Despite the high prevalence of motor comorbidities in 16p11.2 probands, so far, studies have used parent report or global functional measures [17, 32] to assess motor profiles. However, parental reports do not provide detailed characterization of motor performance and lack sensitivity to change needed for outcome studies or clinical trials [15].

Here, we used a comprehensive protocol to compare gait and balance in probands and their unaffected siblings. We identified differences in markers of balance and standardized functional assessments. Children with 16p11.2 mutation demonstrated impaired balance and slower speed during walking and running tasks. Reduced performance on clinical assessments was also identified in parent reports of lower functional abilities using standardized questionnaires. Furthermore, reduced endurance was associated with parents’ responses about their child’s daily activities performance. This study provides initial support of these assessments for use in longitudinal natural history studies and clinical trials in 16p11.2.

Detailed characterization of gait is necessary to quantify the severity and the trajectory of the motor impairments. More importantly, it sheds light on motor pathways that may be involved in known copy number variant genetic conditions such as 16p11.2, the most frequent genetic etiology of ASD. In view of the significant heterogeneity in the cognitive, behavioral, and motor phenotype of children with neurodevelopmental disorders including ASD, the identification of quantifiable features to differentiate their motor phenotypes is highly valuable. Furthermore, this approach may prove useful in new genotype-phenotype studies using motor performance as a target [4].

Impaired locomotion and possibly obesity which was commonly reported among the 16p11.2 group relative to their unaffected siblings may influence their engagement in a range of physical activities and in turn affect their social life and wellness. Furthermore, recent studies focusing on interactions between motor functions and social development in neurodevelopmental disorders highlight increased screen time, obesity, and sedentary lifestyles [19] predisposing these children to other behavioral comorbidities. However, our results using the PEDI-CAT, a useful parent report to measure daily activity function including social and cognitive abilities, may not necessarily capture the entire social domain. In order to better understand the influence of motor function on social development in this population, a more detailed and direct assessment of social skills is needed. Yet, in general, developmental studies point to the benefit of engaging children with motor and cognitive impairments in physical activities in an adaptive way to allow them to thrive socially and physically. Future studies in 16p11.2 and other related disorders will need to be designed to assess more precisely how the motor profile affects the level of physical activity and the risk for these comorbidities.

We recognize the following limitations of this study, including a small sample size of selected families and the use of siblings as control groups instead of unrelated typically developing children. The small subgroup of six probands with a confirmed diagnosis of ASD was not large enough to examine the specific relationship between ASD and motor function; however, it was consistent with the reported 30% prevalence of ASD in 16p11.2 [16, 33]. Our sample of convenience did not allow for studying probands with 16p11.2 duplication and deletion separately. Because there are reported findings showing phenotypic differences between groups, future studies should examine these two genotypic groups separately [6, 16, 17, 34]. In addition, future assessments of fine and gross motor coordination using wearable technologies as well as clinical measures of cognitive and social functions may provide more detailed phenotypic characterization, as well as a better understanding of inter-individual variability. These types of results would, in turn, contribute to the development of more targeted interventions.

## Conclusions

Our study reports quantitative gait measures together with parents’ measures of functionality [10, 35] and as such provides novel characterization of the motor impairments in children with 16p11.2. These findings demonstrate the applicability of our protocol and support its utility to identify and define motor dysfunction in children with neurodevelopmental disorders despite their behavioral challenges.

## Data Availability

The datasets during and/or analyzed during the current study are available from the corresponding author on reasonable request.
